# Albumin Stimulates Epithelial Na^+^ Transport and Barrier Integrity by Activating the PI3K/AKT/SGK1 Pathway

**DOI:** 10.3390/ijms23158823

**Published:** 2022-08-08

**Authors:** Mandy Laube, Ulrich H. Thome

**Affiliations:** Center for Pediatric Research Leipzig, Division of Neonatology, Department of Pediatrics, University of Leipzig, 04103 Leipzig, Germany

**Keywords:** lung, Na^+^ transport, barrier integrity, ENaC, BSA, protein kinase B

## Abstract

Albumin is a major serum protein and is frequently used as a cell culture supplement. It is crucially involved in the regulation of osmotic pressure and distribution of fluid between different compartments. Alveolar epithelial Na^+^ transport drives alveolar fluid clearance (AFC), enabling air breathing. Whether or not albumin affects AFC and Na^+^ transport is yet unknown. We therefore determined the acute and chronic effects of albumin on Na^+^ transport in fetal distal lung epithelial (FDLE) cells and the involved kinase pathways. Chronic BSA treatment strongly increased epithelial Na^+^ transport and barrier integrity in Ussing chambers. BSA did not elevate mRNA expression of Na^+^ transporters in FDLE cells after 24 h. Moreover, acute BSA treatment for 45 min mimicked the chronic effects. The elevated Na^+^ transport was caused by an increased maximal ENaC activity, while Na,K-ATPase activity remained unchanged. Acute and chronic BSA treatment lowered membrane permeability, confirming the increased barrier integrity observed in Ussing chambers. Western blots demonstrated an increased phosphorylation of AKT and SGK1, and PI3K inhibition abolished the stimulating effect of BSA. BSA therefore enhanced epithelial Na^+^ transport and barrier integrity by activating the PI3K/AKT/SGK1 pathway.

## 1. Introduction

Mammalian cells are commonly cultured using media containing fetal serum, such as fetal bovine serum (FBS) and specific supplements depending on the respective cells. Serum provides essential molecules such as hormones, growth and attachment factors, as well as several low-molecular-weight nutrients. Due to the undetermined composition and batch-to-batch variability of serum, the use of serum-free media has emerged. Bovine serum albumin (BSA) is often incorporated as an essential component of most serum-free media. Albumin also represents the major protein in serum, where it makes up approximately 60% of the total protein [1]. Albumin possesses a variety of physiological and pharmacological functions, including the transport of metals, fatty acids, cholesterol, and drugs [1]. Furthermore, albumin is crucially involved in the regulation of osmotic pressure and distribution of fluid between different compartments [2]. Addition of commercial BSA enhanced the growth of human diploid fibroblasts [3]. Moreover, antioxidant functions were attributed to albumin due to free radical scavenging [2]. Albumin has an antithrombotic, anticoagulant effect, possibly because of its capacity to bind nitric oxide (NO), inhibiting its inactivation [4]. Clinically, albumin solutions have been used as volume replacement, and as plasma expanders in a more concentrated form [4]. Albumin is further used experimentally to quantify changes in permeability [5].

Albumin binding proteins were identified on endothelial cells [6], with the receptors gp18 and gp30 involved in endocytosis, and albondin (gp60) mediating transcytosis of albumin through the endothelium [7]. Within the alveoli, the epithelial surface is covered by a fluid layer containing proteins such as albumin, immunoglobulin G, and transferrin. Under physiological conditions, the albumin concentration in the alveolar lining fluid is approximately 10 times lower than that in the plasma, yet during acute respiratory distress syndrome, it rises to plasma levels [8,9]. Albumin clearance from the alveolar space is essential for the recovery from pulmonary edema because increased protein osmotic pressure slows alveolar fluid clearance (AFC) [8]. Specialized membrane surface proteins are involved in albumin uptake from the extracellular environment to the cellular inside. These membrane proteins use clathrin-dependent or dynamin-dependent endocytosis processes. Rat alveolar epithelial cells were shown to express albondin at the cell surface and its activation stimulates an endocytic process that internalizes albondin and membrane-adsorbed albumin [10]. The uptake of FITC-albumin by the alveolar type II (ATII) cell line RLE-6TN was mediated by high- and low-affinity transport systems [11]. Another study suggested albumin, in alveolar lining fluid, to be internalized into ATII and ATI cells via a clathrin-mediated endocytic pathway [9]. In the kidney, megalin and cubilin are involved in the clathrin-mediated endocytosis of albumin from the tubular lumen [12]. Megalin and cubilin expression were also shown in ATII cells [13], and their involvement as receptors for albumin endocytosis has been shown in RLE-6TN cells [11].

AFC is a crucial step in the adaptation of newborns to air breathing. During fetal lung development, the lung is filled with fluid that must be removed before birth. AFC is driven by epithelial Na^+^ transport accomplished by apical epithelial Na^+^ channels (ENaC) and basolateral Na-K-ATPase of ATII cells. Vectorial Na^+^ transport creates an osmotic driving force causing fluid absorption from the air spaces into the interstitium and the bloodstream. Premature infants frequently develop respiratory distress syndrome (RDS) due to structural and functional lung immaturity. Lower expression of ENaC has been demonstrated in premature infants [14], which was associated with decreased AFC [15]. Currently, no treatment to specifically activate Na^+^ transport is available; therefore, substances capable of increasing Na^+^ transport may become useful for RDS-associated morbidity in preterm infants.

How albumin affects alveolar epithelial Na^+^ transport is currently unknown, despite the common use of albumin in cell culture and for AFC measurement as well as its presence in the alveolar lining fluid and association with pulmonary edema. We therefore determined the acute and chronic effects of albumin on Na^+^ transport in fetal distal lung epithelial (FDLE) cells of rats, its effect on barrier integrity, and the involved kinase pathways. We found that albumin strongly stimulates epithelial Na^+^ transport and barrier integrity, and this is achieved at least in part by activation of the PI3K/AKT signaling pathway.

## 2. Results

### 2.1. Effect of BSA on Na^+^ and Cl^−^ Transporters in FDLE Cells

#### 2.1.1. Chronic Effects of BSA

**Figure 1:**
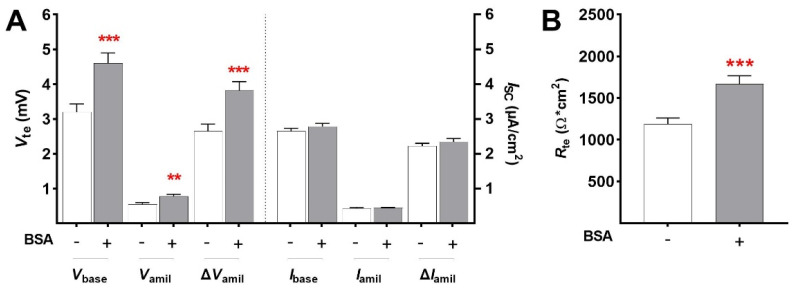
**BSA increases epithelial Na^+^ transport after 24 h.** FDLE cells were cultured in MEM and stimulated with BSA (1.0 g/L) for 24 h. Data are displayed as mean + SEM. (**A**) BSA significantly increased *V*_base_, *V*_amil_, and Δ*V*_amil_ (n = 55–56; ** *p* < 0.01; *** *p* < 0.001 by *t*-test). (**B**) BSA further significantly elevated *R*_te_ in FDLE cells (*** *p* < 0.001 by *t*-test).

We addressed the chronic effect of BSA (1.0 g/L) on epithelial Na^+^ transport in FDLE cells after 24 h. Ussing chamber measurements demonstrated that BSA treatment strongly increased baseline *V*_te_ (*V*_base_: 3.20 ± 0.23 mV to 4.60 ± 0.29 mV; mean ± SEM; Figure 1A; *p* < 0.001), the amiloride-insensitive *V*_te_ (*V*_amil_: 0.55 ± 0.05 mV to 0.78 ± 0.06 mV; *p* < 0.01), and the amiloride-sensitive *V*_te_ (Δ*V*_amil_: 2.66 ± 0.08 to 3.82 ± 0.25; *p* < 0.001). Baseline equivalent *I*_SC_ (*I*_base_), the amiloride-insensitive equivalent *I*_SC_ (*I*_amil_), as well as the amiloride-sensitive equivalent *I*_SC_ (Δ*I*_amil_) were not affected by BSA incubation (Figure 1A). However, the measurements were conducted using current clamp mode, wherein the equivalent *I*_SC_ (eq*I*_SC_) is calculated from the measured potential difference (*V*_te_) and *R*_te_. This means that calculation of eq*I*_SC_ is non-informative under conditions in which both the *V*_te_ and *R*_te_ are altered by the treatment. Indeed, BSA increased *R*_te_ of FDLE cells (1187 ± 73.8 Ω·cm^2^ to 1667 ± 98.5 Ω·cm^2^; Figure 1B; *p* < 0.001). These measurements thus demonstrated that chronic BSA treatment for 24 h strongly increased epithelial Na^+^ transport and barrier integrity.

**Figure 2:**
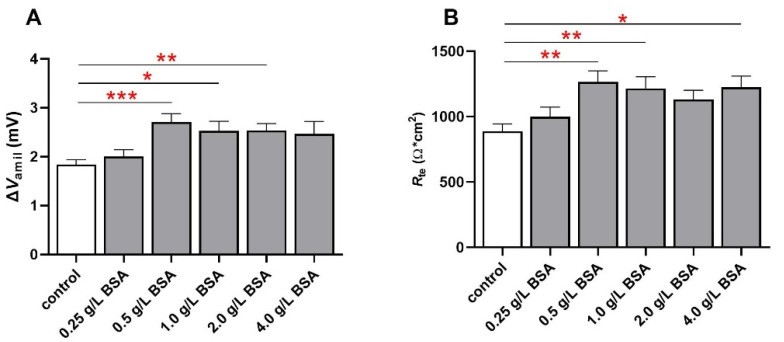
**Concentration-dependent effect of BSA.** BSA increases epithelial Na^+^ transport after 24 h in a concentration-dependent manner. FDLE cells were cultured in MEM and stimulated with BSA (0.25 to 4.0 g/L) for 24 h. Data are displayed as mean + SEM. (**A**) BSA significantly increased Δ*V*_amil_ (n = 57–61; * *p* < 0.05; ** *p* < 0.01; *** *p* < 0.001 by one-way ANOVA with Dunnett’s post hoc test). (**B**) BSA further significantly elevated *R*_te_ in FDLE cells (* *p* < 0.05; ** *p* < 0.01 by one-way ANOVA with Dunnett’s post hoc test).

Next, we addressed a possible concentration-dependent effect of BSA on epithelial Na^+^ transport and barrier integrity. Starting at a concentration of 0.5 g/L BSA, a significant increase in Δ*V*_amil_ was observed (1.84 ± 0.10 mV to 2.71 ± 0.17 mV; Figure 2A; *p* < 0.001). Increasing BSA concentrations above 0.5 g/L did not further elevate Δ*V*_amil_, although Δ*V*_amil_ was still significantly higher at 1.0 g/L and 2.0 g/L compared to control FDLE cells without BSA (Figure 2A; *p* < 0.05; *p* < 0.01). Similarly, a significant increase in *R*_te_ was observed at 0.5 g/L BSA (888.7 ± 55.2 Ω·cm^2^ to 1265 ± 85.9 Ω·cm^2^; Figure 2B; *p* < 0.01), which did not increase further by raising BSA concentrations above 0.5 g/L. Thus, a concentration of 0.5 g/L BSA is sufficient to elevate the epithelial Na^+^ transport and barrier integrity.

**Figure 3:**
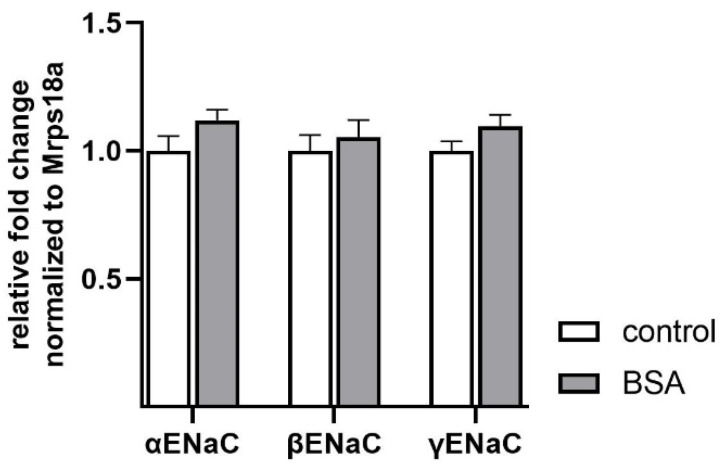
**BSA does not affect ENaC mRNA expression.** FDLE cells were cultured in MEM and stimulated with BSA (1.0 g/L) for 24 h. Data are displayed as mean + SEM normalized to control values (n = 8).

We subsequently tested whether BSA treatment altered mRNA expression of ENaC, which represents one pathway by which BSA might elevate epithelial Na^+^ transport. However, BSA (1.0 g/L) did not affect mRNA levels of α-, β-, or γENaC (Figure 3). Therefore, BSA did not stimulate Na^+^ transport by elevating ENaC mRNA expression in FDLE cells after 24 h.

#### 2.1.2. Acute Effects of BSA

**Figure 4:**
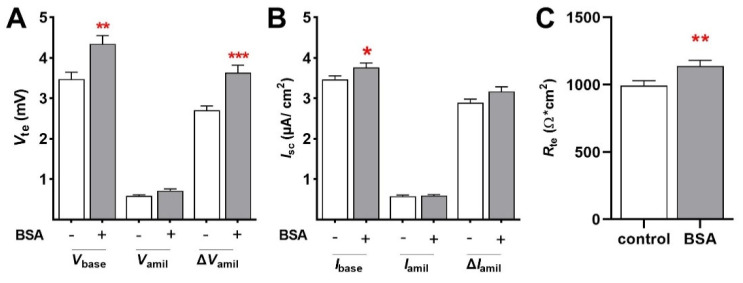
**BSA increases epithelial Na^+^ transport after 45 min.** FDLE cells were cultured in MEM and stimulated with BSA (1.0 g/L) for 45 min. Data are displayed as mean + SEM. BSA significantly increased (**A**) *V*_base_, Δ*V*_amil_, and (**B**) *I*_base_ (n = 67–69; * *p* < 0.05; ** *p* < 0.01; *** *p* < 0.001 by *t*-test). (**C**) BSA further significantly elevated *R*_te_ in FDLE cells after 45 min (** *p* < 0.01 by *t*-test).

Since mRNA expression of ENaC was not affected by BSA, we addressed the acute effect of BSA (1.0 g/L) on epithelial Na^+^ transport in FDLE cells after 45 min. Ussing chamber analyses demonstrated that BSA treatment significantly increased *V*_base_ (3.48 ± 0.17 mV to 4.35 ± 0.20 mV; mean ± SEM; Figure 4A; *p* < 0.01) and Δ*V*_amil_ (2.70 ± 0.11 to 3.63 ± 0.19; *p* < 0.01). *I*_base_ was also significantly higher in BSA-treated cells (Figure 4B; *p* < 0.05), while *I*_amil_ and Δ*I*_amil_ were not affected by BSA after 45 min. Furthermore, BSA increased *R*_te_ of FDLE cells (993 ± 37.2 Ω·cm^2^ to 1139 ± 41.3 Ω·cm^2^; Figure 4C; *p* < 0.01). These measurements thus demonstrated that acute BSA treatment for 45 min was sufficient to increase epithelial Na^+^ transport and barrier integrity.

**Figure 5:**
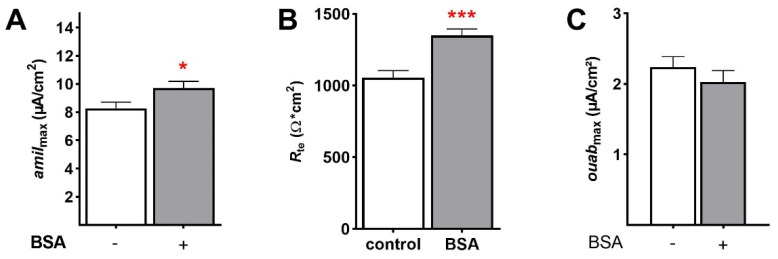
**BSA increases maximal ENaC activity after 45 min, but not that of the Na,K-ATPase.** FDLE cells were cultured in MEM and stimulated with BSA (1.0 g/L) for 45 min. Data are displayed as mean + SEM. BSA significantly increased (**A**) *amil*_max_ and (**B**) *R*_te_ (n = 39–42; * *p* < 0.05; *** *p* < 0.001 by *t*-test). (**C**) In contrast, BSA had no effect on maximal Na,K-ATPase activity (*ouab*_max_) after 45 min (n = 27–31).

Moreover, the maximal amiloride-sensitive apical membrane permeability (*amil*_max_), determined by permeabilizing the basolateral membrane, was significantly increased by BSA (1.0 g/L) in FDLE cells within 45 min (8.28 ± 0.44 µA/cm^2^ to 9.73 ± 0.46 µA/cm^2^; Figure 5A; *p* < 0.05). In addition, BSA strongly increased *R*_te_ within 45 min (1057 ± 48.7 Ω·cm^2^ to 1351 ± 45.1 Ω·cm^2^; Figure 5B; *p* < 0.001). In another set of experiments, the apical membrane was permeabilized loading the Na,K-ATPase with Na^+^, which enables determining the maximal ouabain-sensitive *I*_SC_ (*ouab*_max_). *Ouab*_max_ was not affected by BSA stimulation within 45 min in FDLE cells (Figure 5C). These results suggest that BSA exerts a fast stimulation of epithelial Na^+^ transport by increasing ENaC activity within 45 min, while the activity of the Na,K-ATPase was not altered.

**Figure 6:**
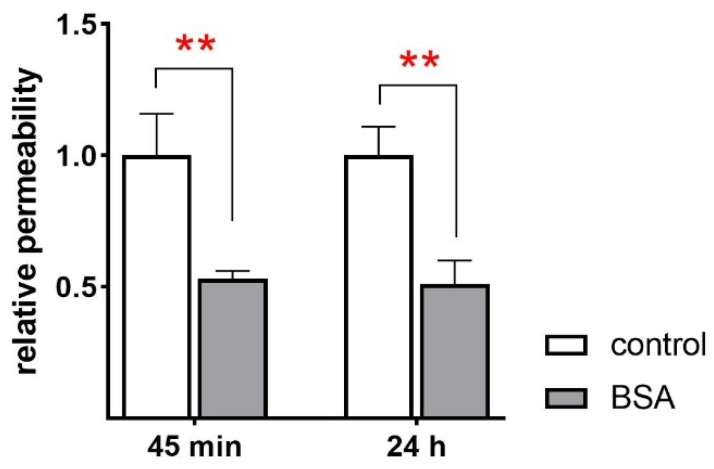
**BSA decreases membrane permeability.** FDLE cells were cultured in MEM and stimulated with BSA (1.0 g/L) for 45 min and 24 h. Data are displayed as mean + SEM normalized to control values. BSA significantly decreased membrane permeability (n = 11–12; ** *p* < 0.01 by *t*-test).

In the previous analyses, BSA exerted a strong effect on barrier integrity, as shown by the significantly increased *R*_te_ after acute and chronic BSA treatment. This was verified by measuring membrane permeability with a FITC dextran assay. BSA-treated FDLE cells exerted significantly lower membrane permeability compared to control cells (Figure 6; *p* < 0.01). This reduced membrane permeability was observed after 45 min and 24 h BSA treatment and confirmed the increased barrier integrity determined in Ussing chambers.

**Figure 7:**
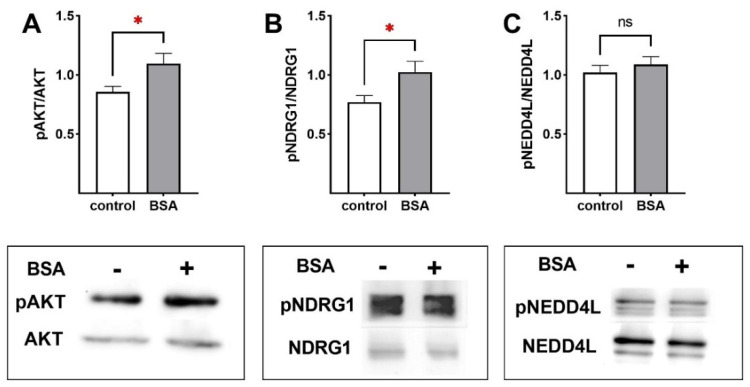
**BSA activates PI3K/AKT/SGK1 signaling pathway.** FDLE cells were cultured in MEM and stimulated with BSA (1.0 g/L) for 45 min. Data are displayed as mean + SEM. BSA significantly increased (**A**) AKT and (**B**) NDRG1 phosphorylation (n = 11–13; * *p* < 0.05 by *t*-test). Western blots of pAKT and total AKT resulted in bands of 60 kDa. Western Blot of pNDRG1 and total NDRG1 resulted in bands of 46 and 48 kDa. (**C**) Western blot of pNEDD4L and total NEDD4L resulted in bands of 110 and 135 kDa (ns = non-significant).

To elucidate possible signaling pathways stimulated by BSA, Western blot measurements were performed determining phosphorylation of several kinases involved in ENaC activation. Phosphorylation of the AKT (protein kinase B) was significantly higher after BSA treatment for 45 min compared to controls (Figure 7A; *p* < 0.05). Moreover, phosphorylation of NDRG1 (n-myc downregulated gene 1) was significantly increased by BSA, indicating an elevated SGK1 (serum- and glucocorticoid-regulated kinase 1) activity (Figure 7B; *p* < 0.05). In contrast, phosphorylation of the ubiquitin ligase NEDD4L (neural precursor cell expressed, developmentally downregulated protein 4-2) was not affected by BSA (Figure 7C). Taken together, these data suggest a stimulation of the PI3K (phosphatidylinositide 3-kinases)/AKT/SGK1 pathway induced by BSA. 

**Figure 8:**
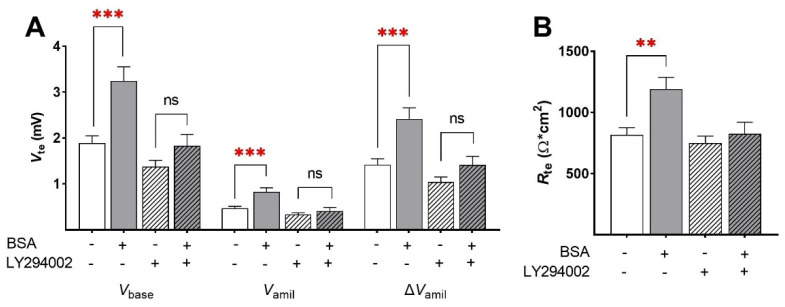
**PI3K inhibition prevents the BSA-induced increase in Na^+^ transport.** FDLE cells were cultured in MEM, stimulated with BSA (1.0 g/L) and LY294002 (10 µM) for 24 h. Data are displayed as mean + SEM. (**A**) BSA significantly increased *V*_base_, *V*_amil_, and Δ*V*_amil_, while LY294002 prevented the BSA-induced increase (n = 39–46; *** *p* < 0.001 by *t*-test; ns = non-significant). (**B**) BSA further significantly elevated *R*_te_ in FDLE cells, which was prevented by LY294002 (** *p* < 0.01 by *t*-test).

Phosphorylation of AKT and SGK1 suggests activation of the respective PI3K pathway by BSA. Whether or not activation of this pathway is responsible for the stimulation of Na^+^ transport by BSA was addressed in Ussing chambers. To this end, PI3K was inhibited by LY294002. As shown before, BSA treatment strongly increased *V*_base_ (1.89 ± 0.16 mV to 3.24 ± 0.31 mV; mean ± SEM; Figure 8A; *p* < 0.001), *V*_amil_ (0.47 ± 0.04 mV to 0.83 ± 0.08 mV; *p* < 0.001), and Δ*V*_amil_ (1.42 ± 0.13 to 2.42 ± 0.24; *p* < 0.001). Furthermore, BSA increased *R*_te_ of FDLE cells (818 ± 57.6 Ω·cm^2^ to 1191 ± 95.5 Ω·cm^2^; Figure 8B; *p* < 0.01). In contrast, inhibition of the PI3K by LY294002 completely abolished the stimulating effect of BSA on epithelial Na^+^ transport and barrier integrity. These measurements thus confirmed that BSA stimulated epithelial Na^+^ transport and barrier integrity by activating the PI3K/AKT/SGK1 pathway.

**Figure 9:**
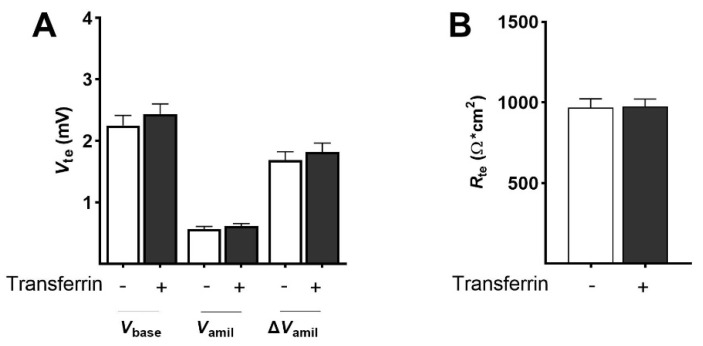
**Transferrin has no effect on epithelial Na^+^ transport and *R*_te_.** FDLE cells were cultured in MEM and stimulated with transferrin (10 µg/mL) for 24 h. Data are displayed as mean + SEM. Transferrin had no effect on *V*_base_, *V*_amil_, Δ*V*_amil_ (**A**) or *R*_te_ (**B**) (n = 39–44).

Finally, transferrin, another serum protein present in the alveolar lining fluid, was analyzed for its ability to stimulate epithelial Na^+^ transport and barrier integrity after 24 h. Ussing chamber analyses demonstrated that *V*_base_ was not significantly altered in transferrin-treated cells (Figure 9A). *V*_base_ of control cells was 2.25 ± 0.16 mV and 2.44 ± 0.16 mV in transferrin-treated cells. In agreement, *V*_amil_ as well as Δ*V*_amil_ were not affected by transferrin. *V*_amil_ of control cells was 0.56 ± 0.04 mV and 0.62 ± 0.04 mV in transferrin-treated cells. Furthermore, Δ*V*_amil_ of control cells was 1.69 ± 0.14 mV and 1.82 ± 0.14 mV in transferrin-treated cells. Regarding *R*_te_, transferrin showed no effect on barrier integrity, with 967 ± 56 Ω·cm^2^ in control cells versus 975 ± 47 Ω·cm^2^ in transferrin-treated cells (Figure 9B). These measurements demonstrated that transferrin treatment for 24 h did not reproduce the effects of BSA on epithelial Na^+^ transport and barrier integrity in FDLE cells.

## 3. Discussion

Albumin is frequently used as cell culture supplement, although knowledge about its cell-specific effects is limited. Whenever we determine effects of hormones or growth factors on epithelial Na^+^ transport, we use BSA-containing medium instead of fetal serum. Control medium lacking BSA yielded lower Na^+^ transport capacity, which led us to determine the effect of albumin on alveolar epithelial Na^+^ transport. We first addressed the chronic effect of BSA in FDLE cells for 24 h that strongly increased epithelial Na^+^ transport and barrier integrity, both by approximately 40%. Furthermore, a concentration of 0.5 g/L BSA was sufficient to elevate epithelial Na^+^ transport and barrier integrity. BSA did not stimulate Na^+^ transport by elevating ENaC mRNA expression after 24 h. Further supporting non-genomic actions, acute BSA treatment for 45 min increased epithelial Na^+^ transport by approximately 25% and barrier integrity by approximately 30%. Moreover, maximal ENaC activity was significantly increased by around 20% within 45 min. In contrast, maximal Na,K-ATPase activity was not affected by acute BSA stimulation. These results suggest that BSA exerts a fast stimulation of epithelial Na^+^ transport by increasing ENaC activity, while the activity of the Na,K-ATPase was not altered. To our knowledge, this is the first study showing a stimulation of ENaC activity by BSA. Overall, no studies addressed any association between cellular ion transport and BSA at all.

There are several regulatory mechanisms known for ENaC stimulation, such as translocation from intracellular pools to the plasma membrane [16,17]. Furthermore, preventing ENaC degradation by phosphorylation of NEDD4L [18] and channel activation by direct phosphorylation [19,20,21] leads to an increased ENaC open probability [19,20]. It was previously shown that AKT increases ENaC activity by phosphorylation of NEDD4L, thereby reducing the affinity of NEDD4L to ENaC [18]. To examine possible signaling pathways stimulated by BSA, Western blots determined phosphorylation of several kinases involved in regulating ENaC activity. Phosphorylation of AKT was significantly higher after BSA treatment. Moreover, phosphorylation of NDRG1 was significantly increased by BSA, indicating elevated SGK1 activity. In contrast, phosphorylation of the ubiquitin ligase NEDD4L was not altered by BSA. Taken together, these data suggest a stimulation of the PI3K/AKT/SGK1 pathway induced by BSA. Whether activation of this pathway is responsible for the stimulation of Na^+^ transport by BSA was analyzed in Ussing chambers. Inhibition of the PI3K completely abolished the stimulating effect of BSA on epithelial Na^+^ transport and barrier integrity. These measurements confirmed that BSA stimulated epithelial Na^+^ transport and barrier integrity by activating the PI3K/AKT/SGK1 pathway. How BSA-activated AKT/SGK1 modulates ENaC activity is yet unknown, since NEDD4L phosphorylation was unchanged. A direct interaction between ENaC and AKT represents another possibility. Recombinant AKT rapidly increased ENaC open probability in outside-out single-channel Patch Clamp recordings of Xenopus laevis oocytes [21]. This rapid response was dependent on phosphorylation of S621 in α-ENaC. Irrespective of the link between AKT and ENaC, activation of receptor tyrosine kinases results in activation of PI3K, followed by formation of phospholipids. These subsequently phosphorylate and activate AKT, resulting in an increased epithelial Na^+^ transport induced by BSA.

Albumin has previously been shown to exert its anti-apoptotic signaling by activating the PI3K/AKT pathway [22]. Similar results were obtained with human serum albumin (HSA) and its anti-apoptotic activity, involving the PI3K/AKT signaling pathway in HUVEC cell lysates [23]. Therein, phosphorylated forms of AKT were detected after HSA stimulation and prevention of HSA protection was achieved with PI3K inhibitors [23]. Albumin endocytosis has been shown to activate the PI3K/AKT pathway, which in a feed-forward mechanism, stimulated albumin endocytosis [24]. Precisely, physiological albumin concentrations (0.01 mg/mL BSA) promoted AKT phosphorylation, which peaked at 15 min [24]. For Western blot analysis, we incubated the FDLE cells with BSA for 45 min and obtained similar results. Albumin is internalized by fluid phase endocytosis mainly through macropinocytosis, also known as “cell drinking” [25]. Macropinocytosis is a nonspecific endocytic pathway that rapidly internalizes very large amounts of plasma membrane and extracellular fluid [26]. It thereby contributes to several physiological processes such as nutrient uptake and nutrient sensing, signaling, antigen presentation, and cell migration [26]. It is initiated by the polymerization of actin at the plasma membrane [26]. Cell types capable of macropinocytosis include dendritic cells, macrophages, B and T cells, epithelial and endothelial cells, fibroblasts, neurons, microglia, and cancer cells [26]. Epithelial cells, for instance, show macropinocytosis in response to growth factors or exposure to pathogens to facilitate their internalization [27]. The remodeling of the cytoskeleton that leads to macropinocytosis requires PI3K activity at the plasma membrane [28]. PI3K inhibitors such as LY294002, which we used to block the BSA effect, are often utilized to inhibit macropinocytosis through inhibition of actin polymerization [26]. The small GTPases Rac1 and Cdc42 as well as p21-activated kinase 1 (PAK1), which are involved in actin polymerization, are further regulators of macropinocytosis [29]. In addition, Ras is suggested to be important in macropinocytosis and can activate PI3K through their Ras-binding domain [30]. Macropinocytosis is differentiated from other types of endocytosis by its unique susceptibility to inhibitors of Na^+^/H^+^ exchange such as amiloride and its analogues such as 5-(N-ethyl-N-isopropyl)amiloride (EIPA) [29]. Inhibition of Na^+^/H^+^ exchangers leads to acidification of the submembranous cytosol with a subsequent failure in recruitment of Rac1 and Cdc42, which inhibits actin polymerization [29]. Notably, macropinocytosis inhibitors such as EIPA inhibited the albumin uptake [31]. In this study, amiloride is used to inhibit ENaC activity that is increased by albumin. With regard to the previous studies, an association between albumin endocytosis, PI3K signaling, and ENaC activity might be conceivable. Moreover, interaction between ENaC and the cytoskeleton through adaptor proteins plays an important role in the regulation of Na^+^ transport [32]. Actin dynamics are mainly mediated by Rho-GTPases such as Rac1, whose signaling pathway was shown to regulate ENaC in the kidney [33]. Additionally, several studies have shown convergence of these pathways, where PI3K promoted Rac1 signaling [34,35] and inhibition of Rac1 also decreased stimulated Na^+^ transport in our FDLE cells [36]. Furthermore, BSA exerted a strong effect on barrier integrity, as shown by the significantly increased *R*_te_ after acute and chronic BSA treatment. Moreover, BSA-treated FDLE cells exerted a significantly lower membrane permeability compared to control cells. This reduced membrane permeability was observed after 45 min and 24 h BSA treatment and confirmed the increased barrier integrity determined in Ussing chambers. These barrier protective effects of BSA have also been unknown to date. Barrier integrity is important for AFC because it prevents airspace flooding that can contribute to edema formation [37].

Finally, another serum protein, transferrin, was used to determine its effect on Na^+^ transport in Ussing chambers. Like albumin, transferrin is also present in alveolar lining fluid and classified as a β-globulin. Similar to albumin, transferrin is considered a negative acute phase reactant and serum levels decrease during inflammatory states [38]. Moreover, transferrin is also commonly used as a cell culture supplement to reduce the amount of animal serum. These similarities led us to analyze transferrin. The concentration of 10 µg/mL used in our analysis was chosen according to published studies using FDLE cells in serum-free culture [39]. In contrast to albumin, transferrin showed no effect on epithelial Na^+^ transport or barrier integrity. As for albumin, we measured the effect of transferrin with a concentration used for cell culture supplement and have not tested other concentrations. We thus cannot rule out that higher concentrations of transferrin might affect Na^+^ transport or barrier integrity.

Taken together, we first showed a stimulation of ENaC activity by BSA. BSA exerts a fast stimulation of epithelial Na^+^ transport by increasing ENaC activity, while the activity of the Na,K-ATPase was not altered. In addition, BSA exerted a strong effect on barrier integrity after acute and chronic BSA treatment and decreased epithelial permeability, confirming the barrier protective effects of BSA. BSA further activated PI3K/AKT/SGK1 signaling within 45 min and inhibition of PI3K prevented the stimulatory effect of BSA. According to the results, we assume that BSA is internalized into FDLE cells by an endocytic process such as macropinocytosis requiring PI3K activity to drive remodeling of the cytoskeleton that, after AKT activation, finally stimulates epithelial Na^+^ transport. The demonstrated properties of albumin highlights pathways that deserve further exploration as targets for alleviating respiratory distress in the future.

## 4. Materials and Methods

### 4.1. Cell Isolation and Culture

All experimental methods were approved by the Leipzig University institutional review board (Landesdirektion Leipzig, permit T07/20, Leipzig, Germany). Sprague–Dawley rats were housed at the Medical Experimental Center of Leipzig University. Rats were kept in rooms with a 12:12 h light–dark cycle, with constant temperature (22 °C) and humidity (55%). Food and water were supplied ad libitum. Pregnant rats were euthanized by Pentobarbital injection at gestational day E20–21 (term E = 22). All methods were carried out in accordance with relevant guidelines and regulations.

FDLE cells, a model of preterm ATII cells, were isolated from fetal lungs as described previously [40]. After mechanical dissociation of fetal lungs, the cell suspension was enzymatically digested by incubation in HBSS (Life Technologies, Darmstadt, Germany) with 0.125% trypsin (Life Technologies) and 0.4 mg/mL DNAse (CellSystems, Troisdorf, Germany) for 10 min at 37 °C and MEM containing 0.1% collagenase (CellSystems) and DNAse for 15 min at 37 °C. FDLE cells were seeded on permeable Snapwell inserts (surface area 1.1 cm^2^, Costar 3407; Corning, Corning, NY, USA) at a density of 10^6^ cells per insert for electrophysiological measurements. For RNA and protein isolation, cells were seeded on larger inserts (ThinCert, #657641, surface area 4.6 cm^2^, Greiner Bio-One, Frickenhausen, Germany) at a density of 2 × 10^6^ cells per insert. The cell culture medium consisted of MEM with 10% FBS, glutamine (2 mM, Life Technologies) and antibiotic-antimycotic (Life Technologies).

### 4.2. Ussing Chamber Measurements

Electrophysiological measurements in Ussing chambers were performed 4–5 days after cell isolation as described before [40]. Equivalent short-circuit currents (eq*I*_SC_) were determined every 20 s by measuring transepithelial voltage (*V*_te_) and *R*_te_ with a transepithelial current clamp (Physiologic Instruments, San Diego, CA, USA) and calculating the quotient eq*I*_SC_ = *V*_te_/*R*_te_. After the eq*I*_SC_ reached a stable plateau (baseline eq*I*_SC_ = *I*_base_), amiloride (10 µM, # A7410, Sigma-Aldrich, St. Louis, MO, USA) was applied to the apical chamber to assess the amiloride-insensitive eq*I*_SC_ (*I*_amil_). The current reduction induced by amiloride (amiloride-sensitive eq*I*_SC_ = Δ*I*_amil_) was used as a measure of ENaC activity. To determine maximal Na,K-ATPases capacity, the apical membrane was permeabilized with amphotericin B (10 µM, # A-4888, Sigma-Aldrich), loading the cytosol with Na^+^. *I*_SC_ was directly measured applying square wave pulses (2 mV, 500 ms) every 5 s with a transepithelial voltage clamp. When *I*_SC_ had risen to its maximum value, ouabain (1 mM, # O3125, Sigma-Aldrich) was added to the basolateral compartment and the ouabain-sensitive component of the amphotericin-induced maximal *I*_SC_ (*ouab*_max_) was calculated. Furthermore, apical Na^+^ permeability was determined by adding amphotericin B (100 μM) to the basolateral compartment. For this experimental setup, 140 mM of basolateral Na^+^ was replaced by 116 mM N-methyl-D-glucamine (NMDG^+^, # M-2004, Sigma-Aldrich) and 24 mM choline, generating a 145:5 apical-to-basolateral Na^+^ gradient. Following amphotericin B addition, when *I*_SC_ had reached its maximum value, the amiloride-sensitive component (*amil*_max_) was determined by adding 10 μM amiloride to the apical compartment. Amiloride and ouabain stock solutions were prepared in water.

### 4.3. mRNA Expression Analyses

Total RNA was isolated using the PureLink RNA Mini Kit (Thermo Fisher Scientific, Waltham, MA, USA) and treated with DNase I (Thermo Fisher Scientific) according to the manufacturer’s instructions. Reverse transcription was carried out employing the Maxima H Minus DNA Synthesis Kit (Thermo Fisher Scientific). Real-time quantitative PCR (RT-qPCR) was performed in the CFX 96 Real-Time system (Bio-Rad, Munich, Germany) using the SYBR Select Master Mix (Fisher Scientific GmbH) and gene-specific primers [41,42] as described before [42]. The resulting molecule concentrations were normalized to a reference gene encoding for the *mitochondrial ribosomal protein S18a* (Mrps18a).

### 4.4. Western Blot Analyses

Western blot studies were performed as described elsewhere [43]. Phosphorylation of AKT was analyzed using antibodies against phospho-AKT at Ser473 (# 9271, Cell Signaling Technology, Inc.) and AKT (# 9272, Cell Signaling Technology, Inc., both kindly provided by A. Garten). Phosphorylation of NDRG1 was used as an assay of SGK1 enzyme activity [44,45,46] and detected with phospho-NDRG1 antibody (# 3217, Cell Signaling Technology, Inc., Danvers, MA, USA) when phosphorylated at Thr346, and NDRG1 antibody (# 5196, Cell Signaling Technology, Inc.). Phosphorylation of NEDD4L was measured with antibodies against phospho-NEDD4L at Ser448 (# 8063, Cell Signaling Technology, Inc.) and total NEDD4L (# 4013, Cell Signaling Technology, Inc.). Suitable secondary antibodies conjugated to horseradish peroxidase (HRP) were used to detect primary antibodies. HRP activity was analyzed by enhanced chemiluminescence (ECL, Amersham, Piscataway, NJ, USA) on X-ray film and band intensity was measured by densitometry using Image-J (National Institutes of Health (NIH), Bethesda, MD, USA). Original Western Blotting images can be found in the supplement.

### 4.5. Cell Permeability Assay

Epithelial permeability was assessed with FDLE cells cultured on Snapwell inserts. After two days in culture, the medium was changed to BSA-containing or control medium. After 45 min or 24 h, the medium was replaced by phenol red-free MEM (Thermo Fisher Scientific) in the lower compartment and phenol red-free MEM containing FITC-dextran (0.25 mg/mL, 3–5 kDa, # 46944, Sigma-Aldrich) in the upper insert compartment. Cells were incubated for another 24 h, followed by analysis of FITC-dextran fluorescence intensity in the lower compartment.

### 4.6. Statistical Analysis

Differences between two groups were analyzed with the unpaired *t*-test. Otherwise, significant differences were determined by ANOVA with Dunnett’s post hoc test. A probability of *p* < 0.05 was considered significant for all statistical analyses. Statistical analysis was performed with GraphPad Prism software (GraphPad Software, La Jolla, CA, USA).

## Data Availability

All relevant data are within the paper.
